# At-line porosity sensing for non-destructive disintegration testing in immediate release tablets

**DOI:** 10.1016/j.ijpx.2023.100186

**Published:** 2023-06-10

**Authors:** Prince Bawuah, Mike Evans, Ard Lura, Daniel J. Farrell, Patrick J. Barrie, Peter Kleinebudde, Daniel Markl, J. Axel Zeitler

**Affiliations:** a University of Cambridge, Department of Chemical Engineering and Biotechnology, UK; b TeraView Limited, 1, Enterprise, Cambridge Research Park, CB25 9PD Cambridge, UK; c Heinrich-Heine-University, Institute of Pharmaceutics and Biopharmaceutics, Dusseldorf, Germany; d Strathclyde Institute of Pharmacy and Biomedical Sciences, University of Strathclyde, Glasgow, UK; e Centre for Continuous Manufacturing and Advanced Crystallisation (CMAC), University of Strathclyde, Technology and Innovation Centre, Glasgow, UK

**Keywords:** Terahertz spectroscopy, Pharmaceutical tablet, Porosity, Disintegration, Real-time release testing, Process analytical technology

## Abstract

Fully automated at-line terahertz time-domain spectroscopy in transmission mode is used to measure tablet porosity for thousands of immediate release tablets. The measurements are rapid and non-destructive. Both laboratory prepared tablets and commercial samples are studied. Multiple measurements on individual tablets quantify the random errors in the terahertz results. These show that the measurements of refractive index are precise, with the standard deviation on a single tablet being about 0.002, with variation between measurements being due to small errors in thickness measurement and from the resolution of the instrument. Six batches of 1000 tablets each were directly compressed using a rotary press. The tabletting turret speed (10 and 30 rpm) and compaction pressure (50, 100 and 200 MPa) were varied between the batches. As expected, the tablets compacted at the highest pressure have far lower porosity than those compacted at the lowest pressure. The turret rotation speed also has a significant effect on porosity. This variation in process parameters resulted in batches of tablets with an average porosity between 5.5 and 26.5%. Within each batch, there is a distribution of porosity values, the standard deviation of which is in the range 1.1 to 1.9%. Destructive measurements of disintegration time were performed in order to develop a predictive model correlating disintegration time and tablet porosity. Testing of the model suggested it was reasonable though there may be some small systematic errors in disintegration time measurement. The terahertz measurements further showed that there are changes in tablet properties after storage for nine months in ambient conditions.

## Graphical abstract

**Figure:**
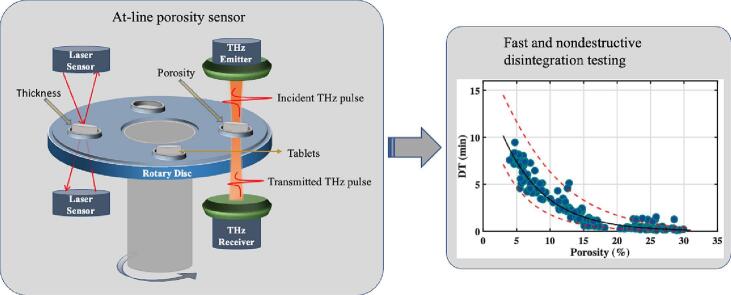

## 1 Introduction

Using relatively small sample sizes of tablets during quality control do not only compromise the quality of products (Markl and Zeitler, 2017) that reach the market but severely restricts product knowledge and process understanding. The regulatory bodies, therefore, introduced the real-time release testing (RTRT) methodology (European Medicines Agency, 2012). RTRT provides a framework based on quality-by-design (QbD) principles (Schlindwein and Gibson, 2017) to ensure the quality of the process stream as well as the final product (Markl et al., 2020; Pawar et al., 2016). RTRT is based on process data and measurements of the critical quality attributes (CQA) captured by appropriate process analytical technology (PAT) tools.

Tablet disintegration and dissolution phenomena are highly complex (Markl et al., 2017a). Advances in measurement techniques that can provide non-invasive and quantitative data motivated several efforts to understand better the disintegration process and its central role in oral drug delivery (Berardi et al., 2021; Jange et al., 2023; Markl and Zeitler, 2017; Quodbach and Kleinebudde, 2015). Specific aspects that have been considered include the mechanism of disintegration; the role of the formulation (e.g., raw material properties); the manufacturing processes (e.g., direct compression, dry and wet granulation, and process settings); the resultant microstructure and bulk tablet properties (e.g., porosity, weight, thickness, and hardness); and the impact of storage conditions (e.g., temperature, humidity) on the disintegration and release behaviour of tablets. Recent studies have identified and classified three main mechanisms that control the overall disintegration kinetics: wettability, dissolution and swelling controlled (Maclean et al., 2021, Maclean et al., 2022; Markl et al., 2021). Specifically, Maclean et al. observed that the disintegration process for formulations based on the binder/filler combination of microcrystalline cellulose (MCC) and lactose was controlled by wettability. It was found that an increase in porosity by 2% can reduce the disintegration time by as much as 77% in such formulations, highlighting the role of porosity as a CQA in immediate release tablets. Hence, a PAT tool is needed to reliably detect subtle changes in porosity that may occur during processing and storage.

The effect of temperature and humidity on the disintegration/dissolution time of tablets has been studied by several researchers using the so-called accelerated stability assessment protocol (Waterman et al., 2007), where tablets are subjected to a range of storage conditions (Chowhan, 1980; Gordon and Chowhan, 2008; Markl et al., 2021; Mollan and Celik, 1995). Depending on the drug's and excipients' chemical and physical stability, the disintegration mechanism can change during storage. These changes have generally been ascribed to moisture sorption and hygroscopicity of the used excipients, which may lead to changes in porosity, tensile strength, and contact angle (Maclean et al., 2022, Maclean et al., 2023). A reduction in the disintegration time due to gel formation, partial dissolution and recrystallisation of soluble fillers (i.e., with high hygroscopicity) upon exposure to high humidity levels have been reported previously (Gordon et al., 1993). However, upon storage at low relative humidity, an increase in disintegration time has been observed, for example, in maltodextrin tablets. It has been argued that partial moisture loss can cause an increase in the intermolecular forces, resulting in stronger, denser, and smaller tablets that significantly contribute to the observed increase in disintegration time (Mollan and Celik, 1995). Aside from the factors mentioned above, the type of particulate deformation during compression can also influence tablet stability during storage. As an example of utilising fillers that undergo brittle fracture (dibasic calcium phosphate dihydrate and lactose monohydrate) as well as those that plastically deform (MCC) during the compression cycle, Sacchetti et al. have reported differences in physical stability during accelerated storage studies (Sacchetti et al., 2017). Rudnic et al. observed that lactose-based tablets exhibited shortening of the disintegration time upon storage for all studied conditions, namely 25 °C/45% RH, 35 °C/60% RH, 45 °C/75% RH (Rudnic et al., 2008) and Maclean et al. reported a decrease in disintegration time as a result of increased porosity for MCC/lactose-based tablets exposed to 30 and 75% RH (Maclean et al., 2022). Marshall et al. conducted a long-term analysis of the impact of storage conditions on the disintegration time of tablets, but apart from this study, there are not many such investigations available in the literature (Marshall et al., 1991). The relative scarcity of studies on the long-term disintegration behaviour of tablets may be due to the destructive, time-consuming nature of existing techniques. The current study provides a non-destructive analysis of the porosity and the long-term disintegration behaviour of immediate release tablets stored under ambient conditions using terahertz time-domain spectroscopy (THz-TDS).

Near-infrared (NIR) (Blanco and Villarroya, 2002; Reich, 2005) and Raman spectroscopies (Cînta Pînzaru et al., 2004; de Veij et al., 2009; Horkovics-Kovats et al., 2022) have been the most popular techniques for real-time monitoring of pharmaceutical unit operations in the industry to date (Kim et al., 2021). However, whilst these techniques can reliably quantify the chemicals in the formulation and dosage form, their ability to yield quantitative predictions on tablet disintegration and dissolution is impaired. This limitation is because the methods primarily rely on surface properties (NIR) or do not have sufficient sensitivity to the physical properties of the porous matrix (Raman). Therefore, multivariate regression methods (chemometric analysis) are required to correlate the observed subtle spectral changes with the respective method to the disintegration and dissolution process for carefully calibrated sets of process conditions and a particular formulation (Freitas et al., 2005; Hernandez et al., 2016; Pawar et al., 2016; Shah et al., 2007).

Generally, for immediate release tablets, porosity/pore structure plays a central role in enabling the transport of dissolution liquid in tablets to activate the disintegration process (Al-Sharabi et al., 2020; Dong and Zeitler, 2022; Markl et al., 2017a, Markl et al., 2018b, Markl et al., 2020; Markl and Zeitler, 2017). It has been demonstrated previously that terahertz spectroscopy can be used as a rapid and non-destructive method to measure tablet porosity reliably (Anuschek et al., 2021; Bawuah et al., 2014, Bawuah et al., 2016, Bawuah et al., 2020; Markl et al., 2017b, Markl et al., 2018a; Moradikouchi et al., 2022). Specifically, THz-TDS is a transmission method to directly measure the total tablet porosity without requiring chemometric analysis and sample preparation (Bawuah et al., 2020).

A recent study showed an excellent correlation between the measured terahertz porosity and the dissolution and disintegration times on a limited number of immediate release tablets prepared using a compaction simulator (Bawuah et al., 2020, Bawuah et al., 2021). In the present study, results are presented on large numbers of immediate release tablets (several thousands) with the aims of understanding errors in the THz measurement and variations in tablet porosity with processing conditions. Both laboratory prepared and commercial tablets are studied. Destructive measurements of disintegration time are then performed in order to develop a predictive model correlating disintegration time with tablet porosity. Furthermore, given the non-destructive nature of the terahertz method, the effect of long-term storage/ageing on porosity and disintegration time has been ascertained via repeated measurements conducted during storage.

## 2 Materials and methods

### 2.1 Laboratory prepared tablets

An immediate-release tablet formulation was prepared with ibuprofen (BLD Pharmatech, Shanghai, China), serving as the active pharmaceutical ingredient (API). The detailed composition of the formulation, which is made up of microcrystalline cellulose (Avicel PH-102, FMC Europe NV, Brussels, Belgium), lactose anhydrous (Supertab21AN, DFE pharma, Goch, Germany), croscarmellose sodium (DuPont Nutrition, Wilmington DE, USA), and magnesium stearate (Fisher Scientific, Fair Lawn NJ, USA), is given in Table 1. To prevent agglomeration, ibuprofen was sieved through a sieve with a mesh size of 1000 μm prior to mixing. The various components were weighed and initially mixed for 10 min at 32 rpm in Bohle mixer LM 20 (LLB Bohle, Germany) without lubricant. Magnesium stearate was then added, and the formulation was mixed for an additional 2 min.

**Table 1:** Chemical composition of the formulation used for all the laboratory-prepared tablets.

Material	% w/w	Quantity [g]
Microcrystalline cellulose	39.1	1564
Lactose anhydrous	46.9	1877
Croscarmellose sodium	3.0	120
Magnesium stearate	1.0	40
Ibuprofen	10.0	399
Total	100.0	4000

The tablets were compacted using a rotary press (Korsch XM 12 Germany) rather than the compaction simulator used in our previous study (Bawuah et al., 2021). The rotary press was fitted with facetted flat-faced punches. Tablets with a diameter of 9 mm were compressed at three different pressures (50, 100 and 200 MPa) and two turret speeds (10 and 30 rpm), resulting in six batches (Table 2). These process conditions are at the limit of the settings that result in high-quality tablets but were chosen to give a wide range of tablet porosities and disintegration times. Additionally, the level of powder lubrication (Delacourte et al., 2008) in combination with the used punch shape (Eiliazadeh et al., 2004; Osamura et al., 2017) can also contribute to tabletting failures under some process conditions. It was, therefore, not surprising to observe occasional chipping on some of the tablets, as shown in Fig. S1 in the Supporting Information. Tablets with noticeable chips were not used in subsequent analyses. Each batch contained 1000 tablets weighing about 400 mg each and a thickness range of 4.4–5.8 mm. The samples were sealed in labelled plastic bags and stored under ambient conditions (about 24 °C and 45% relative humidity).

**Table 2:** Rotary press conditions used to prepare each batch of laboratory-prepared tablets.

Batch name	Turret speed [rpm]	Compaction pressure [MPa]
Batch 1	10	200
Batch 2	30	200
Batch 3	10	100
Batch 4	30	100
Batch 5	10	50
Batch 6	30	50

The first measurement time (labelled M1) was 12 months after the preparation of samples. At this point, the tablets should be fully relaxed mechanically. Unfortunately, the choice of this 12 months initial period was partly out of our control due to the lockdown impact of the COVID-19 pandemic. Firstly 800 tablets from each batch (4800 in all) were analysed by terahertz spectroscopy to gain information on the distributions of porosity. Secondly 30 tablets from each batch (180 in all) were analysed by terahertz spectroscopy followed by destructive disintegration measurements; this formed a training set for a predictive model for disintegration time. Nine weeks later, a further 12 tablets from each batch (72 in all) were analysed by terahertz spectroscopy followed by destructive disintegration measurements; this formed a testing set of the predictive model. A similar protocol was followed nine months later at a second measurement time (labelled M2) to see if there had been any significant changes in the tablets or instrumental stability.

### 2.2 Commercial tablets

Five thousand immediate-release tablets originating from the same production batch of a German generics manufacturer were sourced commercially via a pharmacy. Each commercial tablet was biconvex with an approximate weight of 260 mg, diameter of 9 mm, and thickness of 4.5 mm and contained 100 mg of doxycycline as the active pharmaceutical ingredient. The tablets were uncoated and without embossing. In addition, the formulation contained common excipients like microcrystalline cellulose, lactose monohydrate, magnesium stearate, corn starch, sodium starch glycolate type A, hydrogenated castor oil, and colloidal silica.

Multiple terahertz measurements were made on 16 of the commercial tablets. These repetitions were performed to test reproducibility and quantify the random errors in the technique. Terahertz measurements were then made on the entire set of commercial tablets on two separate occasions to see if ageing impacted results. The first measurement time (labelled M1) was immediately after the tablets were removed from their blister packs. The second measurement time (labelled M2) was after nine months of storage in a plastic container under ambient conditions (about 24 °C and 45% relative humidity).

### 2.3 Terahertz time-domain spectroscopy

A recently developed at-line terahertz sensor (TeraSolve, TeraView Ltd., Cambridge, UK) was used to measure the effective refractive index of almost all the tablets in each batch. Tablets were manually loaded into a carousel for automated measurement. No purging of the sample compartment with nitrogen gas was carried out. With an acquisition rate of 15 Hz, 50 THz waveforms were acquired and averaged for each measurement. Each tablet scan took about 6 s to measure simultaneously the tablet's thickness and the reference and sample terahertz electric field in the transmission configuration. Acquiring a reference measurement is a typical routine where measurement is taken with an empty (atmospheric air composition) compartment, followed by the sample measurements. It is worth mentioning that the current manual tablet loading routine contributes significantly to the 6 s long scanning duration for a tablet. When fully automated, a sub-second scanning time scale can be achieved, which has already been demonstrated in a previous study where porosity of tablets was accurately measured in under one eighth of a second (0.12 s) (Bawuah et al., 2021).

The effective refractive index $n_{\mathrm{eff}}$ was calculated from the measured tablet thickness H, and time of flight difference ∆t between the reference and sample pulses by

(1) $$n_{\mathrm{eff}}=\frac{c∆t}{H}+1,$$

where c is the speed of light in vacuum.

The tablet porosity $f_{\mathrm{THz}}$ was then calculated using the well-established zero-porosity approximation (ZPA) that relates the effective refractive index to the porosity and intrinsic refractive index through the approximation of a linear relationship (Bawuah et al., 2020)

(2) $$n_{\mathrm{eff}}=n_{0}+\left(1-n_{0}\right)f_{\mathrm{THz}}$$

where $n_{0}$ is a parameter (i.e., the intrinsic refractive index of the formulation at zero porosity) determined by calibration with a sample of known porosity (determined by true density measurements using helium pycnometry). Further details regarding the method were described in a previous publication (Bawuah et al., 2021).

### 2.4 Disintegration testing

Disintegration testing on a selected number of laboratory-prepared and commercial tablets was performed using a standard disintegration tester (DT50, SOTAX AG, Switzerland) using the method outlined by the United States Pharmacopeia (USP) chapter 701(USP, 2020). The disintegration endpoints were detected automatically by the unit. Water at 37 ± 2 °C was used as the disintegration medium.

## 3 Results and discussion

### 3.1 Reproducibility and stability testing

To test the reproducibility and stability of the THz instrument, 16 nominally identical tablets from the commercial batch were manually loaded into a carousel auto-feeder. THz measurements were made on each tablet for 120 revolutions of the carousel. The results are shown in Table 3.

**Table 3:** Results of 120 THz-TDS measurements on 16 tablets. The error is the standard deviation of the measurements. The RSD indicates the relative standard deviation of the parameter indicated.

	Thickness H [mm]	RSD (H)	Refractive index $n_{\mathrm{eff}}$	RSD ($n_{\mathrm{eff}}$)
Tablet 1	4.4050 ± 0.0050	0.11%	1.8019 ± 0.0019	0.11%
Tablet 2	4.3809 ± 0.0050	0.12%	1.7867 ± 0.0024	0.14%
Tablet 3	4.4438 ± 0.0069	0.16%	1.8021 ± 0.0026	0.14%
Tablet 4	4.4663 ± 0.0077	0.17%	1.8093 ± 0.0033	0.18%
Tablet 5	4.4316 ± 0.0044	0.10%	1.7984 ± 0.0023	0.13%
Tablet 6	4.4416 ± 0.0056	0.13%	1.8054 ± 0.0022	0.12%
Tablet 7	4.4493 ± 0.0038	0.08%	1.8000 ± 0.0020	0.11%
Tablet 8	4.4062 ± 0.0048	0.11%	1.8072 ± 0.0026	0.14%
Tablet 9	4.3863 ± 0.0040	0.09%	1.7959 ± 0.0023	0.13%
Tablet 10	4.4186 ± 0.0029	0.07%	1.7940 ± 0.0020	0.11%
Tablet 11	4.4434 ± 0.0034	0.08%	1.8047 ± 0.0021	0.12%
Tablet 12	4.3159 ± 0.0040	0.09%	1.8097 ± 0.0017	0.10%
Tablet 13	4.4534 ± 0.0035	0.08%	1.8019 ± 0.0022	0.12%
Tablet 14	4.4125 ± 0.0033	0.07%	1.7979 ± 0.0020	0.11%
Tablet 15	4.4218 ± 0.0036	0.08%	1.7948 ± 0.0018	0.10%
Tablet 16	4.4235 ± 0.0031	0.07%	1.8025 ± 0.0024	0.13%
Average between tablets	4.4188 ± 0.0363	0.82%	1.8001 ± 0.0061	0.34%

These show that repeated measurements on each tablet are very consistent. The thickness measurements for each tablet vary within a narrow range – the relative standard deviations being in the range of 0.07–0.17%. It should be noted that the exact position of the sample within the laser beams that measure thickness will vary each time slightly as the carousel auto-feeder is rotated. Some variations are expected, mainly due to the convex surfaces of the tablet. The effective refractive index, determined by Eq. 1, for each tablet also varies within a relatively narrow range – the relative standard deviations are in the range of 0.10–0.18%.

There is greater variation in thickness and effective refractive index from one tablet to another than the random error of the measurements on a single tablet. The relative standard deviation of the thickness variation between tablets is 0.82%, while the relative standard deviation of the refractive index variation between tablets is 0.34%. Differences in exact thickness between tablets prepared under nominally identical conditions are expected, while some difference in refractive index (because of local porosity differences) is also not surprising, especially for biconvex tablets.

**Fig. 1:**
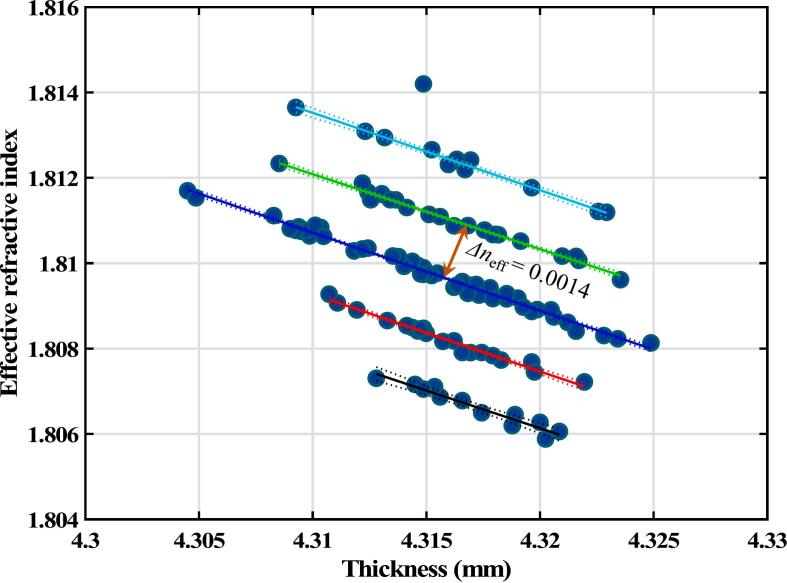
Results from 120 separate measurements on a single tablet.

The fact that the relative standard deviations for the thickness and the effective refractive index on individual tablets are similar suggests that the random error of the thickness measurement is the dominant source of error in determining the effective refractive index. However, a closer examination of the data reveals a more interesting error structure than expected. Fig. 1 shows the calculated refractive index against measured tablet thickness for Tablet 12.

Suppose the thickness measurement is the dominant source of random error. In that case, a linear correlation between the refractive index and measured thickness is expected because the refractive index is calculated using Eq. 1. However, Fig. 1 shows data points falling on five separate parallel lines that do not necessary correlate with the measurement time/sequence. For the data points on each line, random measurements in thickness measurement dominate. Nevertheless, for the data points falling on different lines, it seems that the refractive index varies in increments corresponding to the minimum resolvable refractive index (∼ 0.0014) of the spectrometer arising from the timing measurement. For biconvex tablets, some variation in the refractive index can occur because the exact position of the tablet in the laser beam varies each time slightly as the carousel auto-feeder is rotated. For this tablet, a subtle variation in porosity is observed, as expected for a typical porosity/density distribution of biconvex tablets (May et al., 2013). It is important to emphasise that the artefact described above, i.e., the lines forming in the plot, do not represent actual distinct jumps in refractive index. Similar results to those shown in Fig. 1 were obtained on the other 15 tablets investigated. Overall, the results show that the measurements of refractive index are precise, with standard deviations on a single tablet being about 0.002, with the variation being due to small errors in thickness measurement and from the resolution of the instrument.

### 3.2 Comparison of different batches of laboratory-prepared tablets

Eight hundred tablets from each of the six batches of laboratory-prepared tablets were analysed at measurement time M1 by the at-line terahertz equipment. Thicknesses and effective refractive indices were measured and summarised in Table 4. In a few rare instances (no >1 in each batch of 800), data points were observed that were more than five standard deviations from the mean value (Fig. S2 in the Supplementary Information). These were removed from the analysis as experimental outliers. It is possible that the tablets in these instances had not been manually loaded into the correct position of the carousel auto-feeder.

**Table 4:** Results of terahertz measurements on 800 tablets in each batch. Porosities determined from eq. 2 are also reported. The error is the standard deviation of the measurements that indicates the spread of the distribution. The RSD indicates the relative standard deviation of the parameter indicated. The observed relatively high RSD *f*_THz_ values are inherently due to the lower porosity values of the batches.

Batch	ThicknessH [mm]	Refractive index $n_{\mathrm{eff}}$	RSD $n_{\mathrm{eff}}$	Porosity $f_{\mathrm{THz}}$ [%]	RSD $f_{\mathrm{THz}}$
Batch 1	4.73 ± 0.09	1.784 ± 0.009	0.52%	5.5 ± 1.1	20.2%
Batch 2	4.42 ± 0.17	1.765 ± 0.010	0.54%	7.8 ± 1.1	14.7%
Batch 3	5.03 ± 0.07	1.725 ± 0.011	0.66%	12.7 ± 1.4	10.8%
Batch 4	4.78 ± 0.14	1.695 ± 0.013	0.74%	16.3 ± 1.5	9.3%
Batch 5	5.78 ± 0.05	1.632 ± 0.014	0.88%	23.8 ± 1.7	7.3%
Batch 6	5.25 ± 0.07	1.610 ± 0.016	0.97%	26.5 ± 1.9	7.1%

The relative standard deviations for the effective refractive index were 0.52% (Batch 1) up to 0.97% (Batch 6). However, as discussed in the previous section, the random error in the measurement for a single tablet is lower than this variation (about 0.12%, see Table 3). Therefore, the RSD of every batch is the result of the addition of the random error and the variation within each batch caused by tablets that have different porosities.

The observed higher variation in the case of the lab scale batches, as already mentioned, might be due to various factors such as limited powder flowability and lubrication as well as the punch shape (Delacourte et al., 2008; Eiliazadeh et al., 2004; Osamura et al., 2017) that caused the edges of some tablets to break (chipping) within the batches (see Fig. S1 in Supporting Information). It is also important to emphasise that the lab scale batches were compacted by spanning the compression limits of the tablet press in terms of pressure and speed to test the detection limits of the terahertz method. Compressing tablets at relatively low or high pressures can yield tablets with inconsistent properties within a batch.

**Fig. 2:**
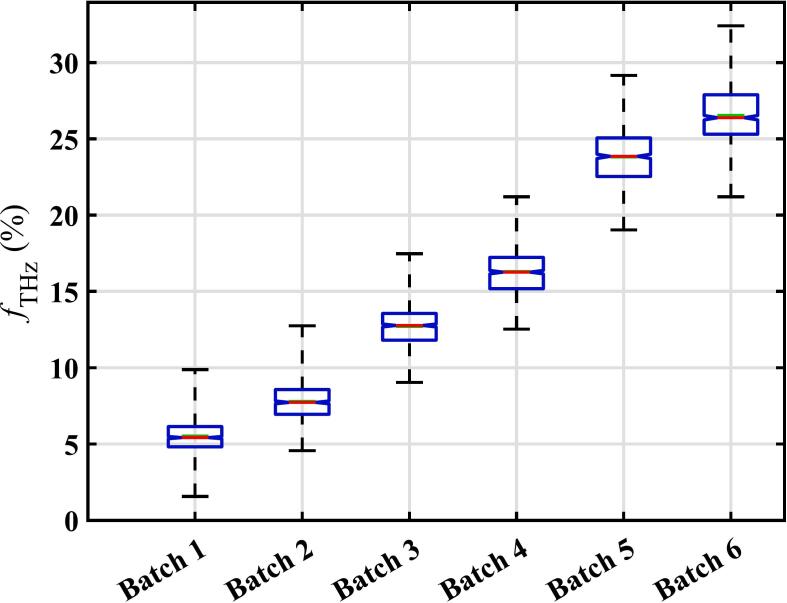
Boxplots showing the distribution of the porosity within each batch. The boxes show the interquartile range, and the whiskers show the full range. The preparation conditions for the different batches are given in Table 2.

The porosities of each tablet were then determined using Eq. 2 with $n_{0}$ = 1.81 obtained by using a set of tablets prepared from same formulation with measured true density, *ρ*_true_ = 1.439 g cm^−3^ using helium pycnometry (Bawuah et al., 2021). Note that inaccurate determination of $n_{0}$ will give a systematic error in the porosity values, though correct trends in values will still be observed. The functionality of eq. 2 is such that the relative standard deviation of porosity values is far larger than the relative standard deviation of refractive index values (particularly when the porosity is low). The resulting porosities are shown in Fig. 2 in boxplot format.

It is clear that the rotary press conditions greatly affect the porosity. As expected, the tablets compacted at the highest pressure (batches 1 and 2) have far lower porosity than those compacted at the lowest pressure (batches 5 and 6). However, it can also be seen that the turret rotation speed also has a significant effect – faster rotation speeds give higher porosity (Batches 2, 4 and 6 compared to Batches 1, 3 and 5). At high turret speed there are problems with filling the tableting dies consistently due to the powder flow limiting the homogeneity of the fill. Batches compressed at high turret speed were relatively thin as well as having a higher porosity (Table 4). This meant they must also have relatively low mass. This was confirmed by weighing 30 tablets from each batch – the average weights were 406, 401 and 411 mg for batches 1, 3 and 5, but 370, 369 and 361 mg for batches 2, 4 and 6. Since the tablet press is displacement controlled, the relative low mass recorded at higher turret speeds means the tablets were compressed under low pressure relative to those compressed at slow turret speed (see Table S1 in the Supplementary Information). The width of the porosity distribution for each batch, as quantified by the standard deviation (see Table 4) or the interquartile range (see Fig. 2), is mainly determined by the compaction pressure with lower pressure giving a wider spread of porosity values. Turret rotation speed has a minor effect, with faster speed giving a slightly wider spread of values.

**Fig. 3:**
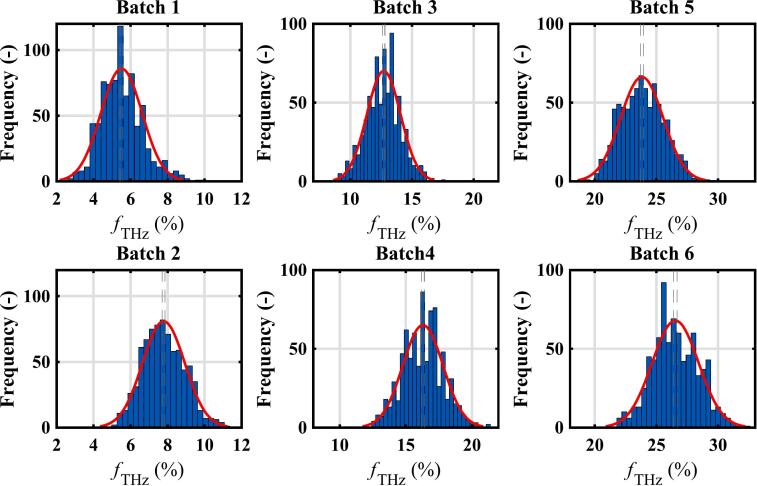
Histograms showing the distribution of porosities for tablets in batches 1–6. The preparation conditions for the different batches are given in Table 2.

Fig. 3 shows histograms of the porosity distribution and a normal distribution fit for each batch. These show that the experimental results have approximately normal distributions. However, data analysis indicates that the distribution is not exactly normal for the tablets prepared at the highest pressure (Batches 1 and 2) and the lowest pressure (Batches 5 and 6). In these cases, the skewness is positive – it is possible to see that there are more samples in the tail at high porosity than in the tail at low porosity. The Shapiro-Wilk test for normality confirmed deviations from normality (see Table S2 in Supporting Information).

### 3.3 Measurement and prediction of disintegration time for laboratory prepared tablets

The disintegration of tablets is affected by porosity, binders, lubricants and hardness. The disintegration performance of microcrystalline cellulose/lactose-based tablets has been classified as limited by wettability (Maclean et al., 2021, Maclean et al., 2022). Porosity is expected to be a major factor affecting disintegration time for the samples investigated in this paper.

The time taken for tablets to disintegrate is generally believed to be hard to measure reliably, particularly for immediate release tablets. The fact that the measurement is destructive means that no checks for reproducibility or error analysis can be performed. The disintegration tester used in this work automatically determines electrically the time taken for the tablet to break down into small particles. It does not require a visual judgement of an operator.

Because of the inherent limitations of conventional disintegration measurements, this work aims to investigate a predictive model for disintegration time based on the terahertz porosity measurements. Forty-two tablets were randomly chosen from each of the six batches. Thirty tablets in each batch formed a training set, while the remaining 12 tablets in each batch formed a validation set.

**Fig. 4:**
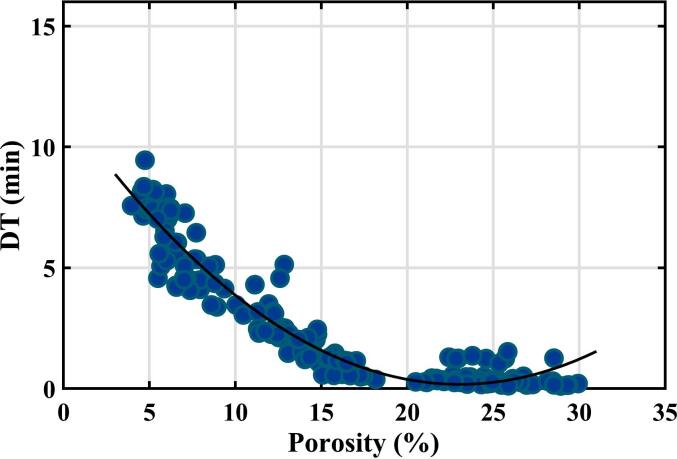
Results of disintegration time (DT) measurements on the training set of samples plotted as a function of tablet porosity. The curve shows a quadratic fit to the data.

Fig. 4 shows a plot for the training set of samples of measured disintegration time against the tablet porosity determined by the terahertz method discussed above. It can be seen that tablets within the training set have a full range of porosities (from 4 to 30%) while the range of disintegration times was 0.11–9.45 min.

Two different methods were used for curve fitting. In Fig. 4, the curve shows the results of fitting to a quadratic equation by ordinary least squares fitting based on our previous publication (Bawuah et al., 2021). The coefficient of determination R^2^ is 0.929 and the RMSE is 0.73 min.

The choice of a quadratic equation is empirical in nature and allows for an increase in disintegration time at very high porosity levels, which is consistent with the literature (Bawuah et al., 2021). At such high porosity values, additional factors such as swelling, hydrophilicity, wetting time and possible gel formation significantly affect the disintegration process. It is helpful to keep in mind that such high porosities are not commonly used for immediate release tablets but rather for orodispersible tablets (ODT) where other excipients are typically used that may not form gels.

The ordinary least squares regression assumes that there are negligible random errors in the terahertz measurements and that there are significant random errors in the disintegration measurements, both of which are good assumptions based on the discussion above. However, the regression also assumes that the random errors in disintegration time have a normal distribution with constant standard deviation. This is certainly not the case at high porosity values because the measured disintegration time cannot become negative.

**Fig. 5:**
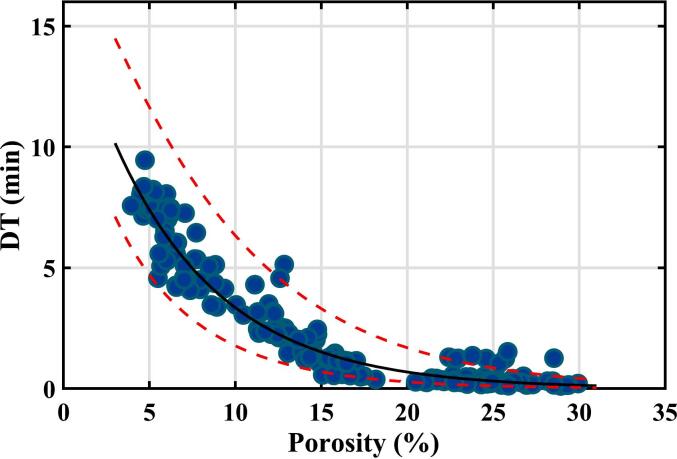
Results of disintegration time measurements on the training set of samples plotted as a function of tablet porosity. The curves show a weighted exponential decay fit to the data and a 95% prediction band using this functionality.

An alternative curve fitting was also investigated using an exponential decay function. For ordinary least squares regression of a linearised exponential decay, it was found that the residuals roughly fell within an envelope that was proportional to the square root of porosity. Assuming this functionality for the standard deviation of random error of disintegration time, i.e., $∆\left(\ln\text{time}\right)\propto \surd \text{porosity}$, enabled weighted least squares regression to be performed on the linearised equation (see Fig. S3 in Supporting Information), and the results then transformed back into the non-linear form. This simulation is shown in Fig. 5. A 95% prediction band is also shown using this assumption for random errors. The prediction band takes into account the uncertainty in the fitted parameters and the random error in an individual measurement. It would be expected that 95% of future measurements would lie within the prediction band region. This second choice of curve fitting, like the quadratic fit, is empirical and has no obvious physical rationale. It was performed because it has the advantage that both the fitted curve and the 95% prediction band exclude negative values of disintegration time.

One aspect of the experimental results worth noting is that the disintegration time observed was usually rapid for the samples with porosities above 20%. However, in eight cases, it was over 1 min. Because the disintegration time measurements are automatic, it is not known whether these eight cases are true values – in which the samples disintegrated slower than expected – or reflect the limitations of the automated testing apparatus when disintegration times are short. Nevertheless, these data points impact the regression results and are one reason the weighted regression scheme in Fig. 5 was performed.

**Fig. 6:**
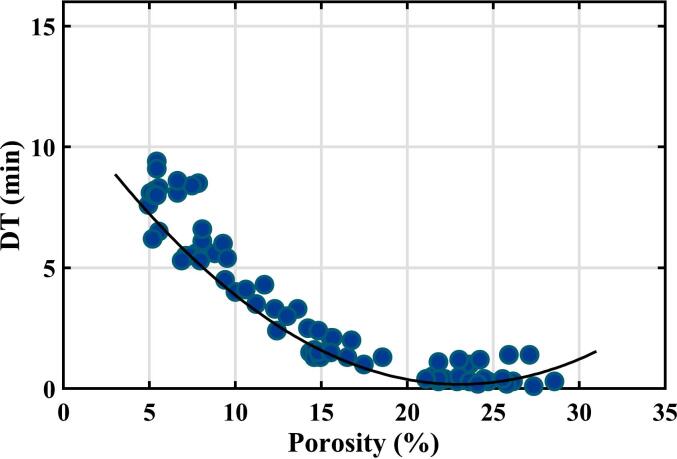
Results of disintegration time measurements on the training set of samples plotted as a function of tablet porosity. The curve shows the quadratic fit curve from Fig. 4 derived from the training set of samples. The equivalent plot using the exponential fit curve from Fig. 5 is shown by Fig. S4 in Supporting Information.

The predictive power of the correlation between disintegration time and terahertz porosity measurement was then tested on the validation samples (12 samples from 6 batches giving 72 data points). The disintegration time results for the validation set and the curve fitting from only the training set of data are shown in Fig. 6.

While the validation set shows the same trends as the testing set, there is a systematic difference between the data sets, with the testing set having longer disintegration times than expected based on both the curve fitting models described earlier. A statistical test using the chi-squared distribution rejected the possibility that the validation set results could come from a population with the quadratic curve fit shown (*p*-value = 2 × 10^−6^).

Unfortunately, the disintegration measurements of the training and validation samples were not made at the same time – there was a 9-week gap between them. This suggests that there must have been a systematic error in the disintegration time measurements. The validation set results agree well with the training data set results if there was a change in the systematic error of 0.6 min (36 s) between the automated measurements on the two sets of samples. A change in systematic error for the porosity measurements between training and validation samples can be excluded because they were randomly chosen from samples that were analysed by the terahertz methods that were performed on the same day. The disintegration apparatus automatically starts timing the measurement when the water reaches the set-point temperature of 37 °C. It is possible that variations in the temperature of the disintegration medium during the experiment occurred. Ideally, the temperature is constant at 37 °C to mimic the body temperature; however random variations ranging between 36.3 and 37.5 °C were observed. Even though there exist a systematic error, the magnitude of error, from quality control point of view, is relatively small and as to whether a tablet disintegrates 36 s faster or slower will have negligible impact on decisions concerning batch quality.

**Fig. 7:**
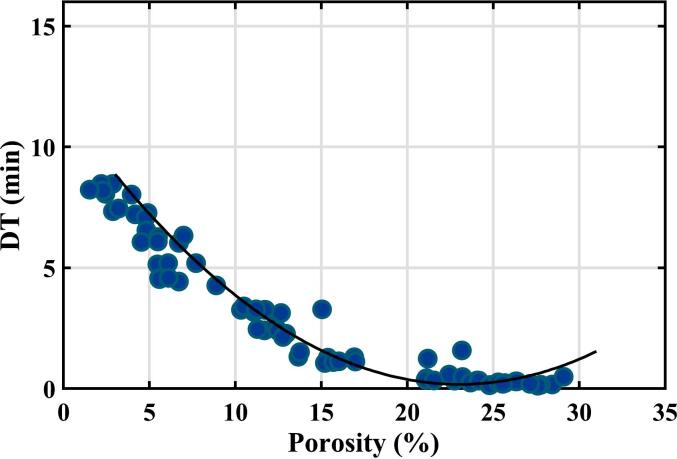
Results of disintegration time measurements as a function of tablet porosity at measurement time M2, 9 months after the original measurements. The porosity values were remeasured by the terahertz technique in case there had been any significant change in properties. The curve shows the quadratic fit curve from Fig. 4 derived from the training set of samples at measurement time M1. The equivalent plot using the exponential fit curve from Fig. 5 is shown by Fig. S5 in Supporting Information.

To test the impact of ageing, terahertz measurements were made on the remaining samples in all six batches nine months after the original measurements to determine the porosity in case there had been some change after storage for this time in ambient conditions. Disintegration times were then measured on 12 tablets from each batch, giving 72 samples in all. The results are shown in Fig. 7.

The results show reasonable agreement with the correlation determined nine months previously. The p-value on whether this data could come from a population with the quadratic curve shown is 0.051. However, visual inspection shows a clear systematic difference between the experimental results and prediction at low porosities. In this case, an agreement would be observed if there had been a change in the systematic error of −0.35 min (21 s), possibly reflecting the disintegration apparatus's limitations.

Overall, the terahertz results suggest that the disintegration measurements of immediate release tablets have a random error of 1.5 min (two standard deviations), together with a possible systematic error of about 0.5 min and hence the effect is not significant.

It is interesting to compare the results of terahertz measurements of porosity at measurement times M1 and M2, separated by nine months after an initial storage period of 12 months (Table 5). These show that there had been some change in porosity values, particularly for batches 1 and 2, suggesting that storing tablets at ambient conditions in an uncontrolled atmosphere does lead to a change in properties but that such changes need not systematically affect the properties in a detrimental fashion. The M1/M2 difference shown in Table 5 appears to change randomly. Existing studies have shown that under ambient conditions the grade of MCC, Avicel PH 102 can absorb up to 5% *w*/w of moisture (Faroongsarng and Peck, 1994; Mihranyan et al., 2004). Given the different porosity levels, the amount of moisture absorbed and dehydrated during long-term storage might differ from batch to batch, which can possibly lead to the observed randomness in the porosity change at M1/M2.

**Table 5:** Results of terahertz measurements of porosity on 800 tablets in each batch at two different measuring times M1 and M2 separated by 9 months. The error bar is the standard deviation of the measurements – note that this is not an “error” but an indication of the spread of the distribution.

	Porosity at M1 f [%]	Porosity at M2 f [%]	Difference [%]
Batch1	5.5 ± 1.1	7.0 ± 1.0	+1.5
Batch 2	7.8 ± 1.1	6.2 ± 1.1	−1.6
Batch 3	12.7 ± 1.4	13.1 ± 1.3	+0.4
Batch 4	16.3 ± 1.5	14.6 ± 1.5	−1.7
Batch 5	23.8 ± 1.7	23.1 ± 1.7	−0.7
Batch 6	26.5 ± 1.9	25.5 ± 1.9	−1.0

### 3.4 Measurements on commercial tablets

**Fig. 8:**
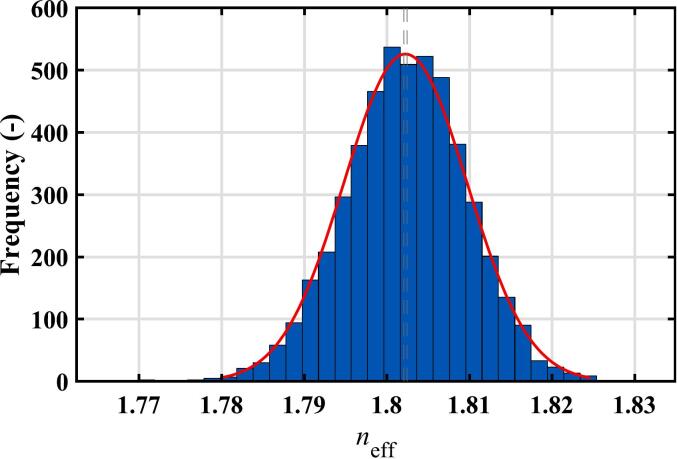
Histogram showing the distribution of effective refractive index for the commercial tablets.

The results of the terahertz measurements on 5000 commercial samples are given in Table 6. The samples are biconvex rather than flat, but the thickness measurements vary less from tablet to tablet than the laboratory-prepared samples. Similarly, the refractive index shows less variation from sample to sample than the laboratory-prepared samples, with a relative standard deviation of 0.4% compared to the range of 0.5–1% for batches 1–6 of the laboratory-prepared tablets. The refractive indices have a normal distribution, as shown in Fig. 8, which is also true after nine months of storage in ambient conditions.

**Table 6:** Summary of terahertz results at two separate times on 5000 commercial tablets.

	Thickness H [mm]	Refractive index $n_{\mathrm{eff}}$	RSD $n_{\mathrm{eff}}$
M1: fresh tablets	4.49 ± 0.04	1.802 ± 0.007	0.4%
M2: Nine months later	4.47 ± 0.04	1.799 ± 0.007	0.4%

Unfortunately, it was not possible to convert the refractive indices into porosity for the commercial tablets. This is because we had no detailed information regarding the exact composition of the formulation of the tablets, and so it is not possible to determine the parameter $n_{0}$ in Eq. 2. As an example of the likely spread of porosities, if $n_{0}$ was 1.94, then the average porosity would be about 15% with a standard deviation of 0.7%. This is a far narrower porosity range than for the laboratory samples and is what would be expected for a commercial product. Our measurements suggest that the manufacturing process for these tablets is reliable.

**Fig. 9:**
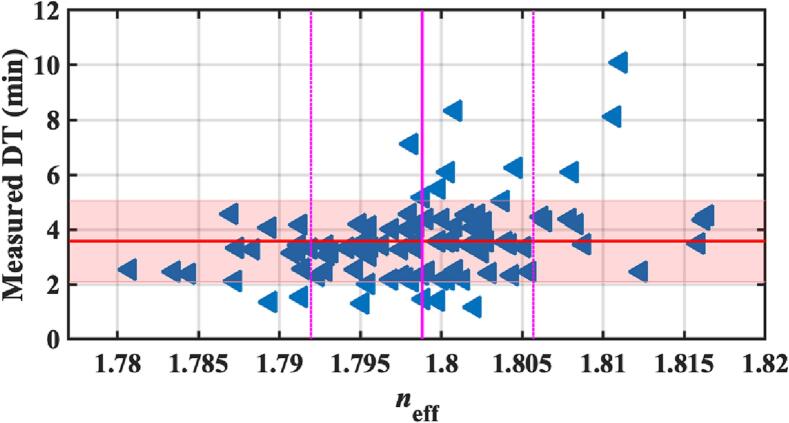
Measured disintegration time verse the effective refractive index of 96 selected tablets from the commercial batch. The solid red line indicates the mean DT with the shades area representing the standard deviation. (For interpretation of the references to colour in this figure legend, the reader is referred to the web version of this article.)

Although it is impossible to determine porosities for these tablets, a correlation between disintegration time and refractive index is expected. After the terahertz measurement, disintegration testing measurements were made on 96 randomly selected tablets. The results are shown in Fig. 9. An extensive spread of disintegration times is observed, notwithstanding the narrow range of refractive indices, and there is no apparent correlation. However, the disintegration apparatus did not perform reliably for these samples. Excipients in the tablets caused the formation of an oily residue layer on the metallic mesh inside the instrument basket. This layer of oil prevented the conducting elements embedded in the apparatus's perforated plastic disk from making contact with the conducting mesh in the basket. Therefore, we suspect the reported disintegration times of >5 min are unreliable. It was unfeasible to determine the endpoint of the disintegration testing visually due to the formation of a cloudy dispersion upon contact of the tablets with the disintegration medium, as this made it impossible to see the tablet during the disintegration experiment. It may be the case that inferring disintegration times from terahertz measurements of porosity for this type of sample are more reliable than attempting direct measurement of disintegration time.

**Fig. 10:**
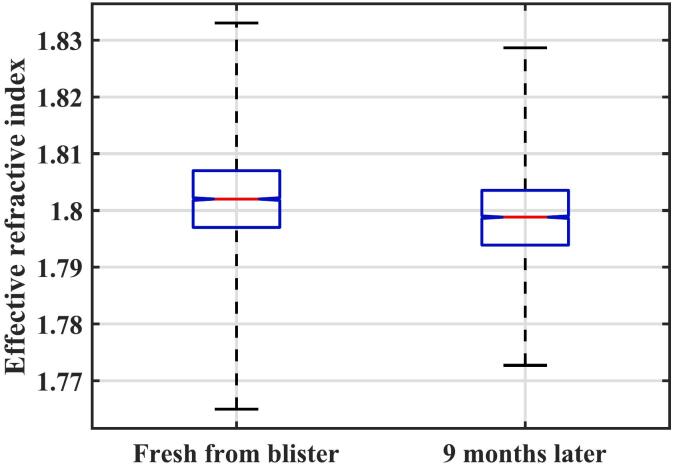
Comparison of the refractive index distributions at two measurement times showing a change in the mean refractive index.

Terahertz measurements were also made on the commercial tablets after nine months of storage time. While the refractive index distributions overlap, there is a small but significant reduction in the average value after storage. This can be seen in Fig. 10 and was confirmed by a statistical *t*-test (*p*-value <10^−6^). This suggests there is a small increase in porosity during the storage time which is consistent with literature (Maclean et al., 2021, Maclean et al., 2022; Markl et al., 2021). It is likely that hygroscopic ingredients in the tablet absorb water and cause some premature swelling of microcrystalline cellulose (Maclean et al., 2022) resulting in the so-called disintegrant pre-activation during storage (Berardi et al., 2021).

### 3.5 Other benefits and current limitations of the terahertz method

**Fig. 11:**
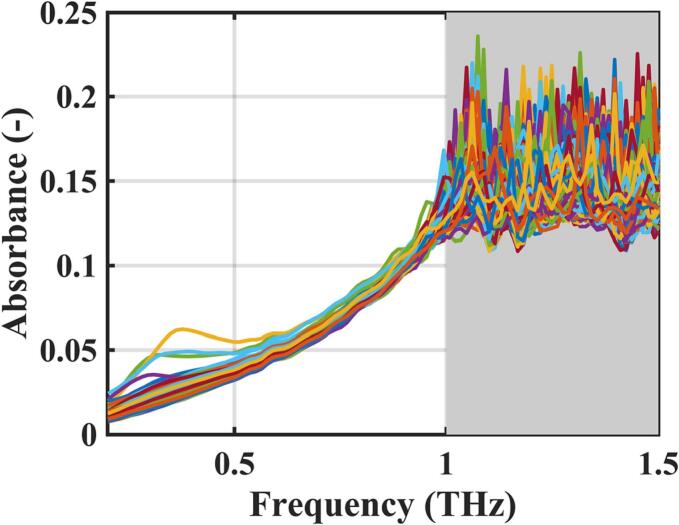
Absorbance spectra of all tablets of Batch 3 compressed at 10 rpm and 100 MPa. The grey shaded area indicates the noisy part of the spectra at high frequencies above 1 THz. The features at frequencies below 0.5 THz observed for a small number of tablet samples are artefacts related to the positioning of the samples and are shown here only for illustrative purposes. There are no known spectral features in organic molecular crystals at such low frequencies.

An added merit to terahertz time-domain spectroscopy, compared to other spectroscopic methods, is the ability to simultaneously measure both the real and imaginary part of the complex refractive index; for example, Fig. 11 shows the absorbance spectra for Batch 3 of the lab scale tablets. In principle it is possible to measure content uniformity or any changes in crystallinity or crystalline solid form in the same measurement data acquired for the porosity measurement. Tracking possible variations in drug content or form change could therefore be achieved by identifying and quantifying the intensity of a prominent spectral fingerprint of the API in the terahertz region. However, the accessible spectral range is quite narrow when measuring through whole tablets (here 0.1–1.0 THz) and spectral features may be located at higher frequencies, e.g., lowest frequency spectral feature of ibuprofen is located at 1.5 THz, so outside the accessible range (King et al., 2011). The dynamic range in THz-TDS is frequency dependent and the high frequencies get attenuated more quickly as highlighted by the grey shaded portion in Fig. 11. The tablets used in this study are, hence, too absorbing to access higher frequencies. It is important for readers to note that the above limitation is sample dependent, and the method should work very well for low absorbing samples or for APIs that possess strong absorption features at lower terahertz frequencies within the accessible range.

The terahertz measurement probes a volume of about 2 mm in diameter through the centre of the tablet in axial direction, hence the porosity is a representation of central portion of the tablet but not the entire tablet volume. This can result in some limitation for tablets that may exhibit significant density distribution in the radial direction. Such density distribution may result from the uneven pressure distribution due to the punch geometry during compression. As a result, most biconvex tablets tend to have lower density, or porosity, in the central volume, which is probed by our method. The low-density region will play a significant role in the disintegration mechanism given that higher porosity results in faster liquid transport and hence faster wicking that activates drug release (Markl and Zeitler, 2017). The ability of the terahertz method to probe this critical portion of biconvex tablets can thus be regarded as an advantage. In principle, it is possible to measure the porosity of the tablets at different locations apart from at the centre of the tablet, but we have thus far restricted our measurement on the central volume.

## 4 Conclusions

We measured the porosity variation of a large number of lab scale and commercially manufactured immediate release tablets using an at-line, non-destructive terahertz sensor. For the lab scale samples, we were able to quantify differences in tablet porosity and determine the impact of these variations on the disintegration performance. Using this approach quality testing based on sampling thousands of tablets rather than a small sample is becoming increasingly feasible, which will open new opportunities for real-time release testing as well for process control in batch and continuous manufacturing alike.

In the case of the lab scale samples, variations in process parameters like compression speed and pressure have shown to significantly influence the porosity and disintegration performance of the output tablets. It is generally observed for the lab scale samples that decreasing compression pressure and increasing compression speed produced tablets with high relative variations in porosity. A predictive model correlating disintegration time with measured porosity was developed. Testing of the model suggested it was reasonable though there may be some small systematic errors in disintegration time measurement.

Comparatively, the manufacturing process of the commercial batch yielded a more consistent product with relative standard deviation in effective refractive index of 0.4% compared to the range of 0.5–1% for the lab scale batches. The standard disintegration testing method struggled to measure disintegration times reliably for the commercial tablets so it was not possible to correlate disintegration time with porosity in this case. However, it may be that rapid non-destructive terahertz measurements are suitable for predicting trends in disintegration times when these are hard to measure by traditional methods.

Capitalising on the non-destructive nature of the terahertz method, we have successfully demonstrated how this method can be used to monitor changes in batch properties due to long-term storage outside the blister pack. By measuring the same batches within a 9-month time gap, the method was able to detect subtle but significant decrease in the mean refractive index. This will in turn cause an increase in the porosity that will possibly impact the disintegration behaviour of the commercial tablets after the nine-month storage time.

The testing of larger number of samples from a population, in conjunction with the ability to monitor ageing related changes in tablets parameters, make it possible to experimentally determine process variation to inform the development of meaningful process models, ultimately resulting in better process and product lifecycle understanding.

## Declaration of Competing Interest

The authors declare that they have no known competing financial interests or personal relationships that could have appeared to influence the work reported in this paper.

## Data Availability

Data will be made available on request.

## Appendix A Supplementary data

Supplementary material Image 1
