# Supplementary material for: At-line porosity sensing for non-destructive disintegration testing in immediate release tablets
Source: Int J Pharm X. 2023 Jun 10;5:100186. doi: 10.1016/j.ijpx.2023.100186 (PMC10314216; doi:10.1016/j.ijpx.2023.100186)
Supplement: Supplementary file 1 — Supplementary material [file mmc1.docx]

*Supporting Information to*

At-line porosity sensing for non-destructive disintegration testing in immediate release tablets

Prince Bawuah^1^; Mike Evans^2^; Ard Lura^3^; Daniel Farrell^2^; Patrick J. Barrie^1^; Peter Kleinebudde^3^; Daniel Markl^4,5^; and J. Axel Zeitler^1^

^1^University of Cambridge, Department of Chemical Engineering and Biotechnology, UK,

^2^TeraView Limited, 1, Enterprise, Cambridge Research Park, CB25 9PD, Cambridge, UK,

^3^Heinrich-Heine-University, Institute of Pharmaceutics and Biopharmaceutics, Dusseldorf, Germany,

^4^Strathclyde Institute of Pharmacy and Biomedical Sciences, University of Strathclyde, Glasgow, UK,

^5^Centre for Continuous Manufacturing and Advanced Crystallisation (CMAC), University of Strathclyde, Technology and Innovation Centre, Glasgow, UK

**Tablet preparation**

Table S1. The turret speed and the mean values for the compaction pressure with the calculated absolute standard deviation.

| Targeted pressure [MPa] | Turret speed [rpm] | Mean pressure [MPa] | Data points |
| --- | --- | --- | --- |
| 50 | 10.4 | 49.72±2.18 | 120 |
|  | 30.4 | 54.25±3.57 | 318 |
| 100 | 10.4 | 106.08±8.02 | 984 |
|  | 30.4 | 91.12±6.48 | 990 |
| 200 | 10.4 | 204.85±12.82 | 978 |
|  | 30.4 | 174.09±16.31 | 1002 |

**Statistical analysis of porosity distributions**

Table S2. Statistical data on the porosities measured for tablets in batches 1-6 (to accompany Figure 3).

|  | Batch 1 | Batch 2 | Batch 3 | Batch 4 | Batch 5 | Batch 6 |
| --- | --- | --- | --- | --- | --- | --- |
| Number of tablets | 800 | 800 | 800 | 800 | 800 | 800 |
| Mean porosity | 5.51 | 7.79 | 12.71 | 16.29 | 23.82 | 26.54 |
| Standard deviation of porosity | 1.12 | 1.14 | 1.37 | 1.52 | 1.73 | 1.88 |
| Relative standard deviation | 20.2% | 14.7% | 10.8% | 9.3% | 7.3% | 7.1% |
| 95% confidence interval for mean | 5.43 5.59 | 7.71 7.87 | 12.61 12.80 | 16.18 16.40 | 23.70 23.94 | 26.40 26.67 |
| 95% confidence interval for standard deviation | 1.06 1.17 | 1.09 1.20 | 1.30 1.44 | 1.45 1.60 | 1.65 1.82 | 1.79 1.98 |
| Skewness | 0.51 | 0.31 | 0.02 | 0.08 | 0.12 | 0.09 |
| Kurtosis^†^ | 3.79 | 3.07 | 2.98 | 2.87 | 2.49 | 2.74 |
| p-value for Shapiro-Wilk test for normality^‡^ | 0.0005 | 0.0007 | 0.29 | 0.19 | 0.0011 | 0.019 |

† The kurtosis for a normal distribution would be 3.

‡ The p-value for the Shapiro-Wilk test gives the probability that the observed or more extreme distribution would occur by chance for a sample assuming that the population does indeed have a normal distribution. P-values below 0.05 are usually taken to indicate a significant deviation from the normal distribution.

**Materials and Methods**


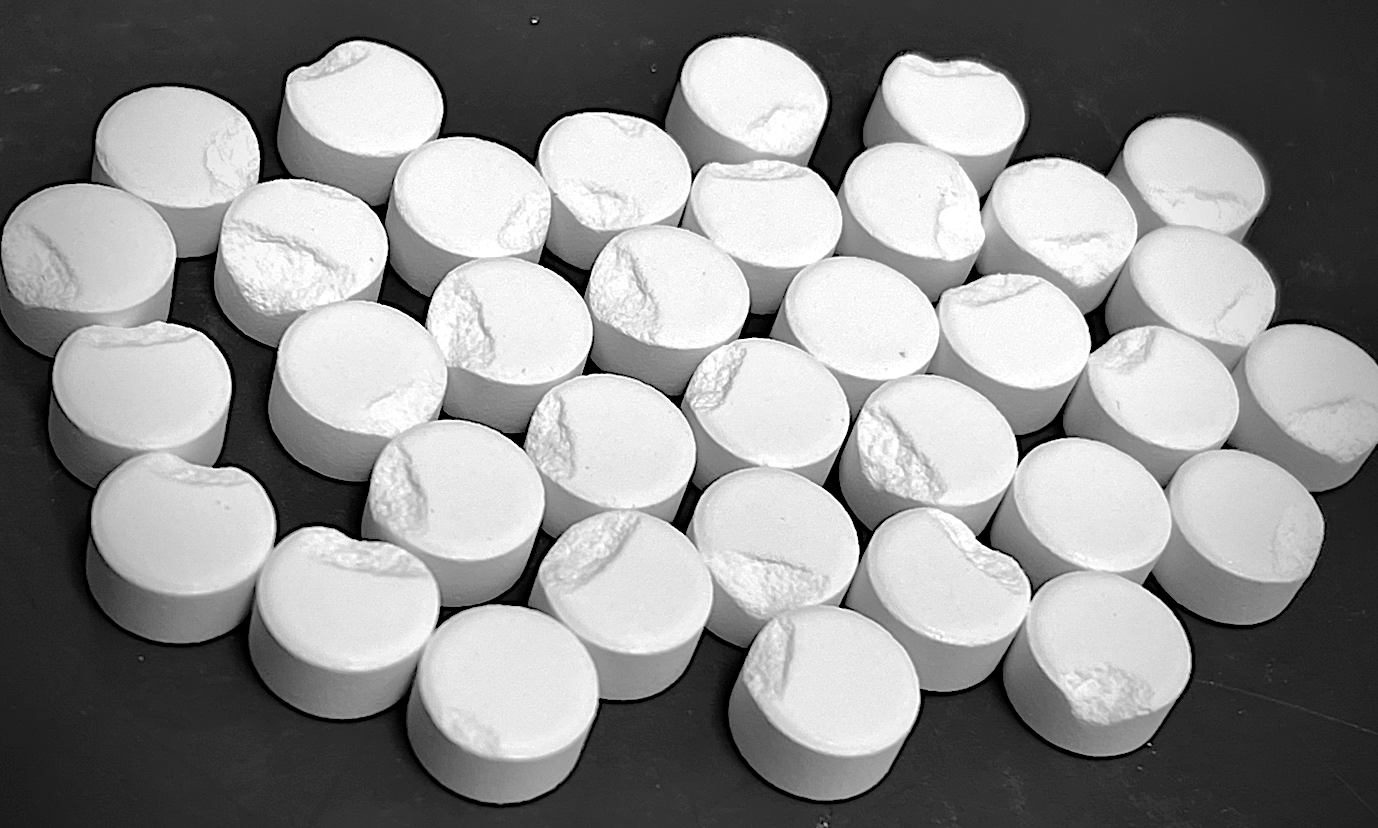


Figure S1. Selected tablets from Batch 3 (compressed at 10 rpm and 100 MPa) showing various degrees of chipping defects during compression.

**Removal of experimental outliers**

Figure S2. Identification and removal of samples that were compromised due to experimental errors from the lab scale batches.

**Modelling the disintegration time for laboratory prepared tablets**

Figure S3. Plot of logarithm of disintegration time against tablet porosity. The error bars in the y-values have been chosen to be proportional to the square root of the x-values. The results of weighted linearised regression with associated 95% prediction bands are shown. This data was then transformed to give Figure 5.

Figure S4. Results of disintegration time measurements on the training set of samples plotted as a function of tablet porosity. The curve shows the exponential fit and prediction band from Figure 5 derived from the training set of samples.

Figure S5. Results of disintegration time measurements at measurement time M2. The curve shows the exponential fit and prediction band from Figure 5 derived from the training set of samples at measurement time M1.
